# Sicherheitsaspekte und Vorbereitung zur Notfallvorsorge und Gefahrenabwehr in Kliniken bei MANV/*Terror*MANV: Ausblick auf zukünftige Herausforderungen anhand von Umfrageergebnissen zur 3. Notfallkonferenz der DGU

**DOI:** 10.1007/s00113-021-01046-y

**Published:** 2021-08-02

**Authors:** Patrick Hoth, Dan Bieler, Benedikt Friemert, Axel Franke, Markus Blätzinger, Gerhard Achatz, AG EKTC der Deutschen Gesellschaft für Unfallchirurgie

**Affiliations:** 1 Klinik für Unfallchirurgie und Orthopädie, Rekonstruktive und Septische Chirurgie, Sporttraumatologie, Bundeswehrkrankenhaus Ulm, Oberer Eselsberg 40, 89081 Ulm, Deutschland; 2 Klinik für Unfallchirurgie und Orthopädie, Wiederherstellungs- und Handchirurgie, Verbrennungsmedizin, Bundeswehrzentralkrankenhaus Koblenz, Rübenacher Straße 170, 56072 Koblenz, Deutschland; 3 Klinik für Orthopädie und Unfallchirurgie, Heinrich-Heine-Universitätsklinikum Düsseldorf, Moorenstraße 5, 40225 Düsseldorf, Deutschland; 4 Akademie der Unfallchirurgie GmbH, Wilhelm-Hale-Straße 46b, 80639 München, Deutschland; 5 Deutsche Gesellschaft für Unfallchirurgie, Berlin, Deutschland

**Keywords:** Kritische Infrastruktur, Traumanetzwerk, Krankenhaussicherheit, Krankenhausalarm- und Einsatzplanung, Sicherheitskonzept, Critical infrastructure, Trauma network, Hospital security, Hospital emergency operations plan, Security concept

## Abstract

**Hintergrund:**

Weltweite terroristische Aktivitäten seit „9/11“ und folgend auch im europäischen Raum haben im Rahmen der Bewertung von kritischer Infrastruktur in Deutschland zu einem Umdenken auch hinsichtlich der Sicherheit an und in Kliniken geführt.

**Ziel der Arbeit:**

Die vorliegende Publikation befasst sich mit der Bewertung vorliegender Konzepte zu Themen wie „Alarmierung“, „Sicherheit“, „Kommunikation“ und „Vorbereitung“ im vorgenannten Kontext.

**Material und Methoden:**

Anhand einer Literatursichtung sowie einer Umfrage unter den Teilnehmern*innen der 3. Notfallkonferenz der DGU (Deutsche Gesellschaft für Unfallchirurgie) werden diese Thematik und die aktuell vorliegende Situation weiter analysiert und vorgestellt.

**Ergebnisse:**

Die gewonnenen Daten verdeutlichen, dass ein Großteil der Kliniken zwar über eine Krankenhausalarm- und Einsatzplanung verfügt, jedoch die Frequenz der Aktualisierungen und die innerklinische Kommunikation zur Steigerung der Wahrnehmung eine deutliche Streuung zeigen.

Weiterhin verdeutlichen die Ergebnisse eine Heterogenität der vorliegenden innerklinischen Alarmierungskonzepte sowie einen Mangel an Sicherheitskonzepten und Kooperationen mit Sicherheits- und Wachdiensten. Zudem zeigt sich, dass die Thematik einer möglichen CBRN(chemical, biological, radiological, nuclear)-Bedrohung in der Risikoanalyse noch nicht adäquat wahrgenommen wird bzw. umgesetzt ist.

**Diskussion:**

Zusammenfassend scheint die latente Bedrohung durch terroristische Aktivitäten dazu geführt zu haben, dass sich deutsche Kliniken in der Bewertung als kritische Infrastruktur mit der Thematik „Krankenhausalarm- und Einsatzplanung“ auseinandergesetzt und diese überwiegend auch umgesetzt haben. Allerdings zeigt sich für die nachgeordneten Bereiche und die aus der Alarmplanung ableitbaren Konsequenzen noch nicht die nötige Stringenz, um letztendlich adäquate Reaktionen in diesen besonderen Szenarien im Hinblick auf die Sicherheit in und an deutschen Kliniken zu gewährleisten.

## Abstract (en)

**Background:**

Worldwide terrorist activities since “9/11” and subsequently also in the European region have led to a rethinking in the context of the evaluation of critical infrastructure in Germany, also with respect to security at and in hospitals.

**Objective:**

This publication deals with the evaluation of existing concepts on topics such as “alerting”, “security”, “communication” and “preparation” in the aforementioned context.

**Material and methods:**

Based on a literature review as well as a survey among participants of the 3rd emergency conference of the DGU (German Society for Trauma Surgery), this topic and the currently existing situation are further analyzed and presented.

**Results:**

The data obtained illustrate that while the majority of hospitals have a hospital alert and response planning, the frequency of updates and intrahospital communication to increase awareness show significant variation. Furthermore, the results illustrate a heterogeneity of the existing intrahospital alerting concepts as well as a lack of security concepts and cooperation with security and guard services. Furthermore, it is evident that the topic of a possible CBRN (chemical, biological, radiological, nuclear) threat is not yet adequately perceived or implemented in the risk analysis.

**Discussion:**

The latent threat of terrorist activities appears to have led German hospitals to address the issue of hospital alarm and response planning in their assessment as critical infrastructure and to have implemented this for the most part; however, the subordinated areas and the consequences that can be derived from alarm planning do not yet show the necessary stringency to ultimately ensure adequate responses in these special scenarios with respect to security in and at German hospitals.

## Hintergrund und Fragestellung

Durch die zunehmende Konfrontation mit terroristischen Aktivitäten seit den Anschlägen vom 11. September 2001 und der daraus erwachsenen Bedrohung für die europäischen Staaten sowie deren Gesundheitssysteme hat das Thema „Sicherheit in deutschen Kliniken“ gleichermaßen an Bedeutung gewonnen [5, 8, 10, 14]. Auf europäischer Ebene müssen diesbezüglich die Terroranschläge von Paris 2015, Nizza 2016 und Berlin 2016 als relevante Katalysatoren genannt werden. Ganz aktuell haben gerade nun zuletzt nochmals die Ereignisse von Nizza und Wien aus dem Jahr 2020 das Thema der ständigen latenten Terrorbedrohung ins Bewusstsein gerufen.

Für Deutschland verdeutlichen Beispiele wie der Terroranschlag in Halle 2019 mit beabsichtigter Stürmung einer Synagoge sowie die Anschlagsserie auf türkische Einrichtungen in Waldkraiburg im Frühjahr 2020, dass derartige Bedrohungen auch in Deutschland bestehen und jederzeit als gegeben angenommen werden müssen. In diesem thematischen Zusammenhang soll die folgende Publikation sich nun näher mit dem Aspekt einer Bestandsaufnahme zum Thema „Sicherheit“ an und in deutschen Kliniken befassen.

Dazu wurde neben einer entsprechenden Literatursichtung weiterhin anhand eines Fragebogens, der Teilnehmer*innen der 3. Notfallkonferenz der Deutschen Gesellschaft für Unfallchirurgie (DGU) vorgelegt wurde, eine weitergehende Erhebung zum Iststand sowie zu Erwartungshaltungen und Vorstellungen zu diesem Thema vorgenommen. Die durchgeführte Bestandsaufnahme zeigt, dass bereits vielerorts strukturiert Maßnahmen zur Steigerung der Sicherheit an und in der Klinik durchgeführt wurden und werden. Allerdings sind individuelle Sicherheitskonzepte noch nicht in jeder Klinik vorhanden. Zudem sind bestimmte Aspekte der vorliegenden Konzepte durchaus zu optimieren (Stichwort: Alarmierung) bzw. neu zu überdenken oder zu implementieren (Stichwort: CBRN [chemische, biologische, radiologische, nukleare] – Bedrohung).

Somit soll die vorliegende Publikation in einem ersten Schritt den Istzustand näher beschreiben und Anregungen und Ableitungen formulieren lassen, denen es sich in Zukunft zu widmen lohnt.

## Einleitung

Sicherheit in den Kliniken ist nicht erst seit den Anschlägen zu Beginn des jungen Jahrtausends (Madrid März 2004, London Juli 2005, Moskau März 2010 und Januar 2011, Brüssel Mai 2014, Paris Januar 2015 und November 2015, Istanbul Januar 2016 und Juni 2016, Brüssel März 2016, Nizza Juli 2016, Berlin Dezember 2016, Istanbul Januar 2017, Manchester Mai 2017 sowie die oben genannten Anschläge aus den Jahren 2019 und 2020) zu einem ernst zu nehmenden Aspekt in der Krankenhausplanung geworden [12]. Ein wesentlicher Anteil der Literatur, die Veränderungen in der Vorbereitung der Klinik auf Großschadenslagen thematisiert, sind Publikationen nach dem 11. September 2001 [18, 22] mit den an diesem Tag stattgefundenen Anschlägen auf das World Trade Center in New York. Während der Schwerpunkt in diesen Arbeiten auf konzeptionellen Ideen wie „emergency plans“ liegt, ist die Thematik „Sicherheit an und in der Klinik“ sowohl qualitativ als auch quantitativ kaum abgebildet.

Lange Zeit hat man eine mögliche und unmittelbare Bedrohung des Gesundheitssystems als solche nicht wahrgenommen, zudem scheint die dokumentierte Fallzahl an Anschlägen auf Krankenhäuser in Europa im Vergleich zu anderen Kontinenten eher gering [11]. Die Analyse der stattgehabten terroristischen Anschläge in europäischen Großstädten mit einem möglichen „second hit“ – Szenario fernab des primären Anschlagsortes – hat hier in der strategischen Planung zu einem Umdenken geführt. Dazu gehört auch die unvermeidliche Wahrnehmung, dass terroristische Vereinigungen und Gruppierungen keinen „humanitären Maßstab“ und „keine humanitäre Hemmschwelle“ mehr an ihre Anschlagsziele legen, sondern mit ihren Mitteln einer Gesellschaft möglichst großen Schaden zufügen wollen [7]. Dabei muss neben mit Sprengstoff bewaffneten Terroristen auch eine mögliche Bedrohung durch biologische, chemisch und nukleare Kampfmittel einkalkuliert werden [17].

Für die präklinische Situation bei einem Terroranschlag besteht offensichtlich eine hohe Gefährdung für die Personen vor Ort, insbesondere auch für die Rettungs- und Einsatzkräfte. Hier besteht teilweise eine unmittelbare Bedrohung durch z. B. den Einsatz von Schusswaffen und explosiven Stoffen sowie eine in der Regel sehr hohe Dynamik vor Ort. Hossfeld et al. konnten diese Aspekte bereits sehr schön darstellen und gerade auch das Konzept der sogenannten unsicheren, teilsicheren und sicheren Zone um einen Anschlagsort und der Relevanz für die Rettungskräfte präklinisch beschreiben [13].

Angesichts der Neubewertung dieser möglichen Bedrohungslagen mit Auswirkung auf das gesellschaftliche Leben und deren Funktionstüchtigkeit werden, gerade z. B. auch nach Ankündigungen und Aufrufen terroristischer Gruppierungen zur Durchführung von Anschlägen eben auch an Kliniken, Krankenhäuser zur kritischen Infrastruktur gezählt und sind damit hinsichtlich ihrer gesellschaftlichen Bedeutung ein potenziell attraktives Ziel für „asymmetrische“ Kräfte, wie eben z. B. terroristische Organisationen. Das Bundesamt für Bevölkerungsschutz und Katastrophenhilfe (BBK) hat hierfür einen Leitfaden zur Identifikation und zur Reduzierung von Ausfallrisiken in kritischen Infrastrukturen des Gesundheitswesens formuliert [6].

Daraus resultierten das Konzept und die Notwendigkeit der Erstellung einer Krankenhausalarm- und Einsatzplanung (KAEP) als Fundament der Sicherstellung der Patientenversorgung und zum Schutz des Krankenhauses [25] bei u. a. terrorassoziierten MANV-Situationen. In dieser Planung sind mitunter die unterschiedlichen Möglichkeiten von Schadenslagen, die Zuweisung von Führungs- und Leitungsverantwortlichkeiten sowie das Zusammenspiel zwischen verschiedenen Behörden beim Auftreten von unterschiedlichen Szenarien zu regeln und stellen somit entsprechende Handlungsanweisungen dar.

Ziel dieser Publikation ist, neben einer entsprechenden Literatursichtung v. a. eine Bestandsaufnahme anhand einer Fragebogenauswertung zu diesem Themenkomplex vorzustellen. Befragt wurden Teilnehmer*innen der 3. Notfallkonferenz der DGU. Inhalte des Fragebogens zielten v. a. auf die Bewertung von Sicherheitsfragen in der eigenen Einrichtung bzw. klinischen Infrastruktur ab. Dabei wurden als Indikatoren sowohl das Vorhandensein einer KAEP als auch Absprachen mit örtlichen Behörden und die Kooperation mit einem Sicherheits- und Wachdienst thematisiert.

## Studiendesign und Methoden

Die Datenerhebung erfolgte anhand eines selbst konzipierten, anonymisierten Fragebogens mit insgesamt 28 Fragen. Die Antwortmöglichkeiten zu den 28 Fragen wurden von ihrer Struktur her in eine Nominal- (17 Fragen) oder eine Ordinalskala (11 Fragen) eingeteilt, mitsamt der Möglichkeit von Einfachantworten. Zu 2 Fragen bestand eine zusätzliche freie Antwortmöglichkeit zur Meinungsäußerung.

Diese wurden 203 Teilnehmer*innen der 3. Notfallkonferenz der DGU an der Berufsgenossenschaftlichen Unfallklinik Ludwigshafen am 29.11.2019 ausgehändigt. Das Publikum der 3. Notfallkonferenz bestand sowohl aus medizinischem Krankenhauspersonal als auch Mitarbeitern*innen der Rettungsdienste, aus der Politik, dem Gesundheitswesen sowie dem Sanitätsdienst der Bundeswehr, wobei alle in Deutschland tätig waren.

Der Fragebogen versuchte, folgende grundsätzliche Themenschwerpunkte zu erfassen:

- Wahrnehmung der Terrorbedrohung,
- Vorbereitung auf mögliche *Terror*MANV-Szenarien,
- Sicherheit an Kliniken.

**Figure 1:**
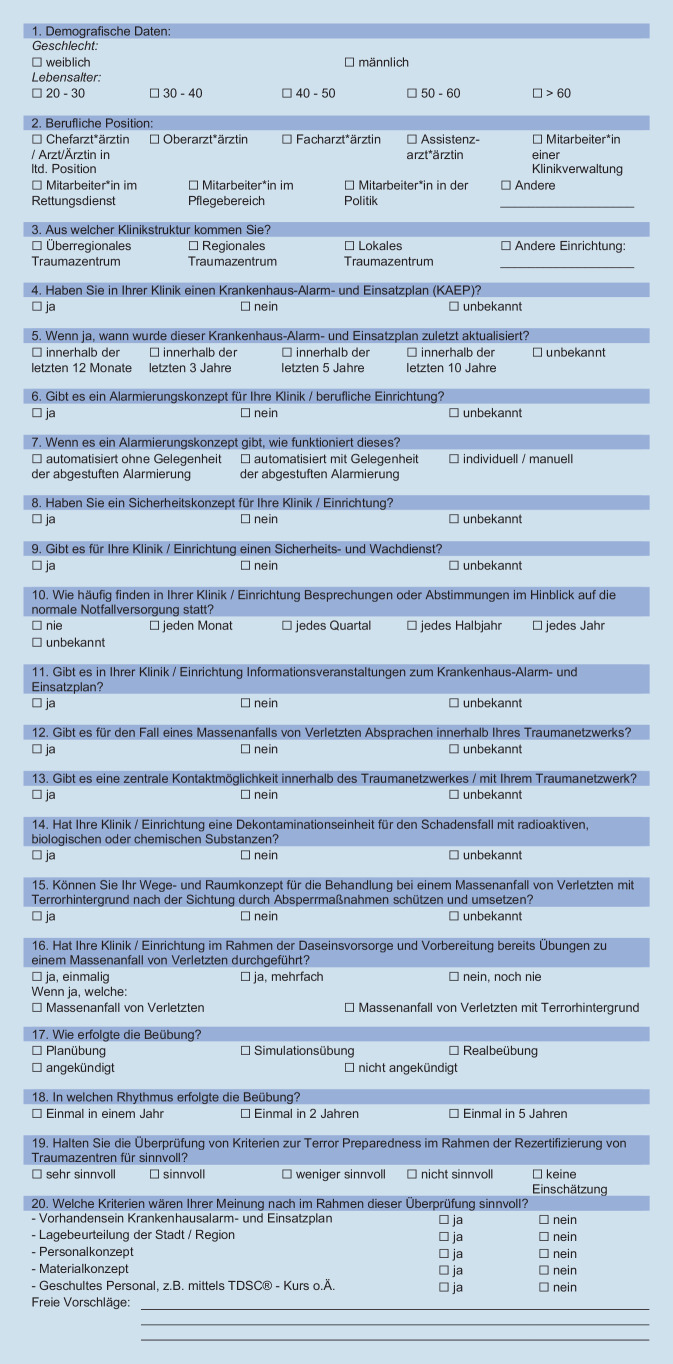

Insgesamt konnten 85 (42 %) Fragebogen für die Auswertung gewonnen werden. Dabei wurden für die vorliegende Arbeit 20 Fragen berücksichtigt, die oben aufgeführte Themenschwerpunkte inhaltlich beantworten konnten (Abb. 1).

Die übrigen 8 Fragen haben sich nicht mit der Thematik „Sicherheit in und an der Klinik“ beschäftigt, sodass diese in der vorliegenden Originalarbeit nicht berücksichtigt wurden, da hier eben unmittelbar kein thematischer Kontext gegeben war.

Primäre Endpunkte unserer Publikation sind:

- das Vorhandensein sowie die Frequenz der Aktualisierung einer Krankenhausalarm- und Einsatzplanung,
- Häufigkeit, Art und Durchführung von Übungen an und in deutschen Kliniken,
- Evaluation der „terror preparedness“ im Sinne einer zu gewährleistenden Daseins- und Notfallvorsorge beim Auftreten von Terrorlagen mit zu definierenden Kriterien,
- bestehende Alarmierungs- und Sicherheitskonzepte in den Kliniken.

Als sekundäre Endpunkte definieren wir:

- demografische Daten der Teilnehmer*innen,
- die Verfügbarkeit eines zentralen Ansprechpartners im jeweiligen Traumanetzwerk,
- das Vorhalten einer Dekontaminationseinheit.

Die erhobenen Daten der Fragebogen wurden in einer Excel-Datenbank (Microsoft®-Excel®, Version 16.47 (2021), Microsoft Corporation, Redmond, WA, USA) ausgewertet und sämtliche Abbildungen der Publikation aus diesem Datensatz generiert.

Fragebogen ohne Angaben einer Antwort wurden bei der Auswertung in der jeweiligen Rubrik nicht berücksichtigt.

## Ergebnisse

Die erhobenen Daten aus 85 Fragebogen stellen aus unserer Sicht eine gute Repräsentanz – sicher jedoch mit gewissen Einschränken – der Traumazentren in Deutschland dar, da wir sowohl eine adäquate Gesamtzahl an Teilnehmer*innen als auch unterschiedliche Funktionsträger*innen aus den verschiedenen Klinikstrukturen befragen konnten. Für die Beantwortung unserer Fragen sehen wir v. a. die hohe Anzahl an leitendem Funktionspersonal (*n* = 56) als relevant an, da dieses Personal sehr häufig in konzeptionelle Planungen an Kliniken miteinbezogen ist. Weiterhin darf davon ausgegangen werden, dass die Thematik der Notfallkonferenz generell Teilnehmer*innen angesprochen hat, die in den entsprechenden Kliniken entsprechenden Themen vertreten. Die Tatsache, dass mit der oben genannten Anzahl an abgegebenen Fragebogen eine Rückläuferquote von 42 % unserer ausgehändigten Fragebogen erreicht konnte, ist durchaus zu hinterfragen, kann jedoch abschließend durch die Autoren auch nicht näher interpretierend beantwortet werden. Trotz mehrfacher Hinweise und Aufrufe zur Unterstützung der Umfrage während der Notfallkonferenz konnte leider nur der genannte Anteil eingesammelt bzw. zurückerhalten werden. Zukünftig muss für derartige Befragungen vielleicht die Ausgabe der Teilnahmezertifikate an die Rückgabe von Fragebogen gekoppelt werden.

Die ersten drei Fragen unseres Fragebogens waren zur demografischen Einordnung der Teilnehmer*innen der 3. Notfallkonferenz konzipiert.

Von den Befragten waren 83 % (*n* = 65) männlich und 17 % (*n* = 13) weiblich. Die Hälfte der Befragten war zum Zeitpunkt der Befragung 50 Jahre und älter (51 %, *n* = 43), ein Viertel jeweils zwischen 40 und 50 Jahre (25 %, *n* = 21) und ein weiteres Viertel zwischen 20 und 40 Lebensjahre alt (24 %, *n* = 24).

**Figure 2:**
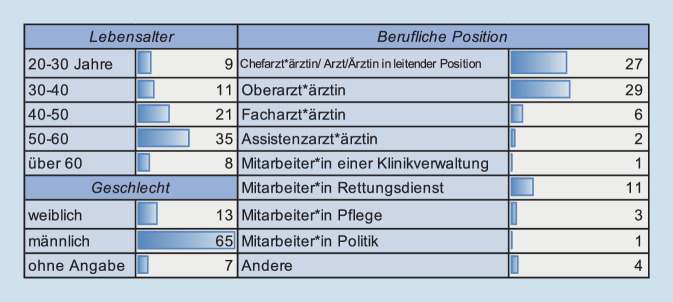

Für die Frage der beruflichen Position definierten wir unterschiedliche Funktionen in den Traumazentren sowie berücksichtigten auch die verschiedenen Tätigkeitsbereiche der Befragten in innerklinischen wie außerklinischen Strukturen. So zeigte sich, dass 32 % (*n* = 27) der Befragten entweder Chefarzt*ärztin in ihrer Klinik oder Ärztin/Arzt in einer leitenden Position waren. Ein weiteres Drittel (35 %, *n* = 29) arbeitet als Oberarzt*ärztin. In geringerer Anzahl nahmen auch Mitarbeiter*innen des Rettungsdienstes (13 %, *n* = 11), aus dem Pflegebereich (*n* = 3), der Klinikverwaltung (*n* = 1) und der Politik (*n* = 1) an der Umfrage teil (Abb. 2).

Da ein KAEP ein zentrales Steuerungselement für eine derartige Notfallsituation ist, zielte eine Frage auf das Vorliegen eines solchen ab. 84 % (*n* = 70) bestätigten das Vorhandensein eines KAEP, während 13 % (*n* = 11) eine solche Planung in ihrer Klinik unbekannt war. Lediglich 2 Befragte (3 %) verneinten die Frage.

**Figure 3:**
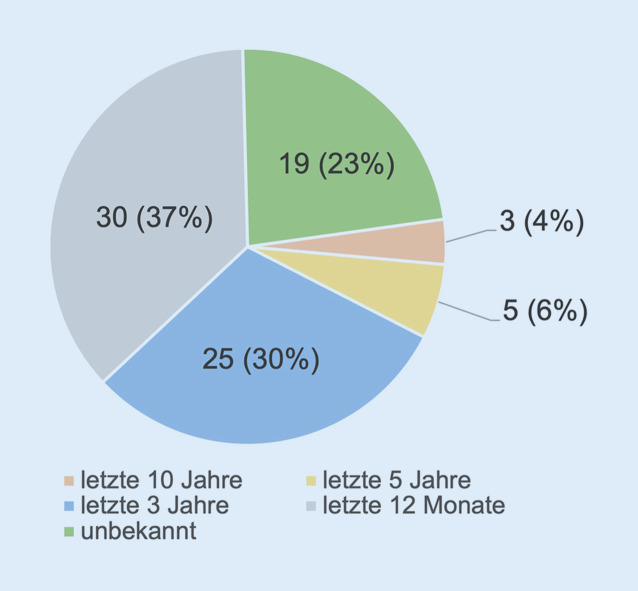

Weiterführend interessant in diesem Zusammenhang erschien der Aspekt der diesbezüglich letzten Aktualisierung des KAEP. Die Mehrheit der Befragten hat innerhalb der letzten 12 Monate eine Aktualisierung des KAEP wahrgenommen (37 %, *n* = 30). Bei weiteren 30 % (*n* = 25) der Befragten erfolgte eine Aktualisierung in den letzten 3 Jahren. 23 % (*n* = 19) konnten keine Aussage zur Aktualisierung treffen, da schlicht unbekannt (Abb. 3).

**Figure 4:**
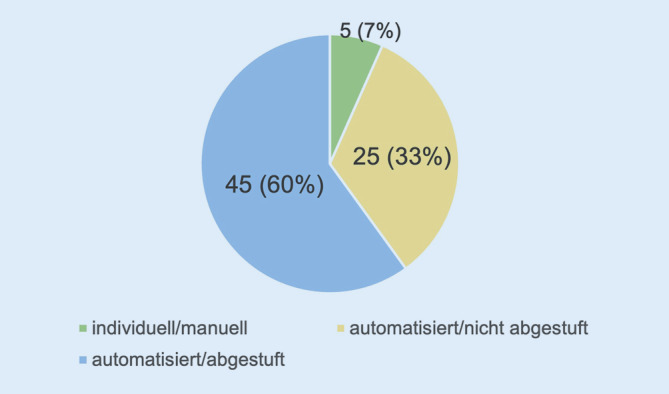

89 % (*n* = 76) der Befragten berichteten über das Bestehen eines Alarmierungskonzeptes in ihren Kliniken, nur 4 % (*n* = 3) haben kein eigenes Alarmierungskonzept. Für den Fall des Vorliegens wurde im nächsten Schritt abgefragt, wie dieses umgesetzt wird. Als Kategorien gaben wir zur Auswahl „automatisiert/nichtabgestuft“, „automatisiert/abgestuft“ und „individuell/manuell“. Knapp über die Hälfte (60 %, *n* = 45) kreuzten die Antwortmöglichkeit „automatisiert/abgestuft“ an, gefolgt an zweiter Stelle von „individuell/manuell“ mit 33 % (*n* = 25). 7 % (*n* = 5) werden „automatisiert/nichtabgestuft“ im Bedarfsfall alarmiert (Abb. 4).

**Figure 5:**
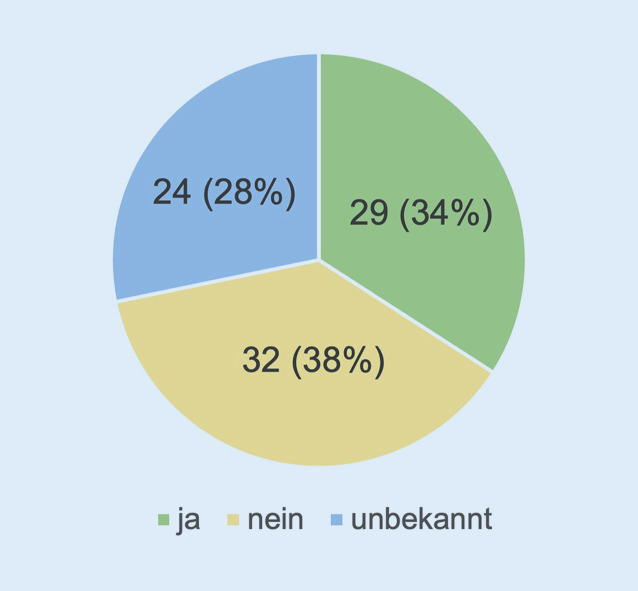

Weiterhin wurden die Teilnehmer*innen zum Vorliegen eines möglichen Sicherheitskonzeptes für ihre Klinik befragt (Abb. 5). Hier zeigte sich in der Auswertung eine ziemlich gleichwertige Verteilung auf die 3 Antwortmöglichkeiten. Das heißt, 34 % (*n* = 29) bejahten das Vorhandensein eines Sicherheitskonzeptes, während 38 % (*n* = 32) dies verneinten. Bei 28 % (*n* = 24) lag die Information hierzu nicht vor, also „unbekannt“.

**Figure 6:**
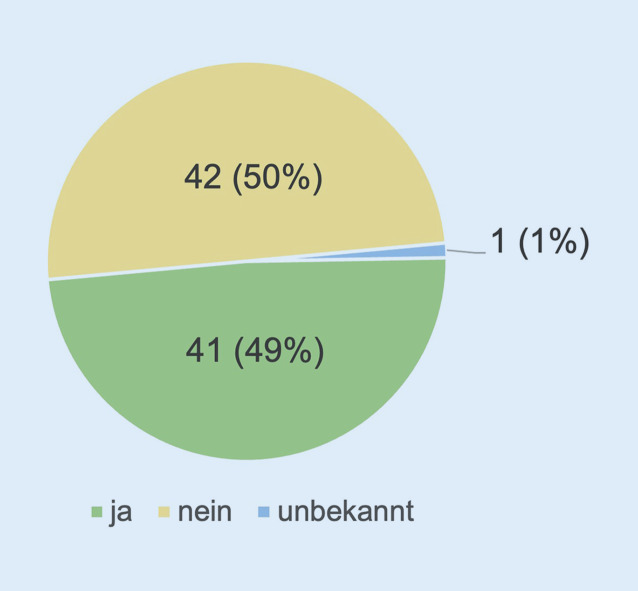

Dabei wurde im Weiteren noch die mögliche Zusammenarbeit mit einem Sicherheits- und Wachdienst abgefragt. 50 % der Befragten (*n* = 42) arbeiten nicht mit einem Sicherheitsdienst zusammen, 49 % (*n* = 41) wiederum verfügen über eine Kooperation bzw. entsprechende Beauftragung (Abb. 6).

Als ein weiteres Element zur Wahrung von Sicherheit in Kliniken darf sicher die innerklinische Kommunikation angeführt werden. Somit wurde auch die Häufigkeit von Besprechungen zur normalen Notfallversorgung in den Traumazentren abgefragt. Die Durchführung derartiger Besprechungen erfolgt bei 38 % (*n* = 32) in jedem Quartal, bei 24 % (*n* = 20) sogar jeden Monat. Wiederum führen 7 % (*n* = 6) bzw. 6 % (*n* = 5) diese Besprechungen zweimal bzw. einmal im Jahr durch, 10 % (*n* = 8) aber auch generell nicht.

Im Hinblick auf die Bekanntmachung der KAEP in den Kliniken bzw. Information über Veränderungen oder Aktualisierungen gaben 49 % (*n* = 42) an, keine Infoveranstaltungen durchzuführen, 46 % (*n* = 39) nahmen dies jedoch regelmäßig vor.

Hinsichtlich der Verfügbarkeit einer zentralen Kontaktmöglichkeit innerhalb des zuständigen Traumanetzwerks zeigte sich folgende Verteilung: 54 % (*n* = 45) verfügen über einen zentralen Ansprechpartner im eigenen Netzwerk, 21 % (*n* = 17) besitzen keine zentrale Kontaktmöglichkeit. Bei 25 % (*n* = 21) konnte die Frage nur mit „unbekannt“ beantwortet werden.

**Figure 7:**
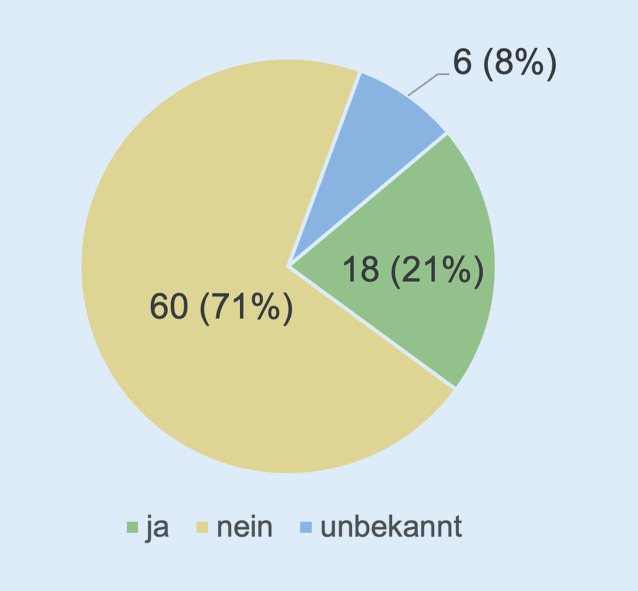

Da gerade auch nach Anschlagsereignissen wie im Falle des russischen Oppositionsführers Alexej Nawalny immer auch Szenarien mit chemischen, biologischen oder nuklearen Kampfstoffen in Betracht gezogen werden müssen, zielte die letzte für diese Publikation berücksichtige Frage auf die apparative Ausstattung mit einer Dekontaminationseinheit ab. Die überwältigende Mehrheit von 71 % (*n* = 60) der Befragten besitzt keine derartige Einheit. Nur 21 % (*n* = 18) verfügen über dieses Element (Abb. 7).

Hinsichtlich der Themenkomplexe „Vorhandensein eines Sicherheitskonzeptes/Absprachen mit Behörden bzw. innerhalb der Traumanetzwerke“ zeigten sich folgende Ergebnisse:

Ein Drittel der Befragten gab an, dass ein Sicherheitskonzept in der eigenen Klinik vorliegen würde (*n* = 29, 34 %), mehr als ein Drittel verneinten dies (38 %, *n* = 32). 24 Befragten war die Information über das Vorliegen eines Sicherheitskonzeptes unbekannt.

Auf die Frage von bereits erfolgten Absprachen mit Behörden im Falle eines MANV zeigte sich eine ähnliche prozentuale Verteilung. Ein Drittel der Befragten verneinte das Vorhandensein von jeglichen Absprachen (*n* = 32, 38 %), weniger als ein Drittel bestätigten vorhandene Absprachen (*n* = 20, 24 %). Die Mehrheit der Befragten äußerte wiederum eine Unkenntnis (*n* = 33, 39 %).

**Figure 8:**
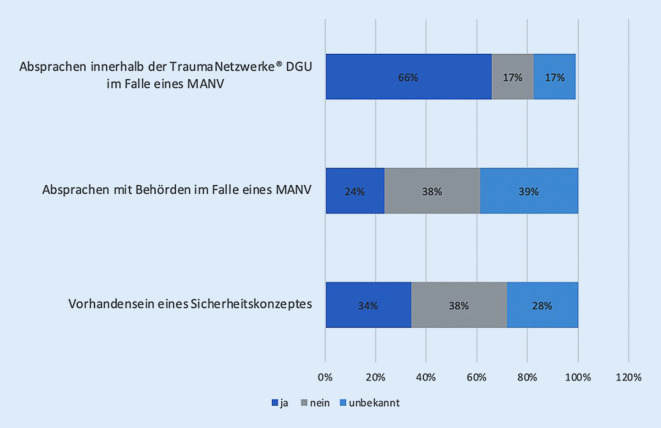

Etablierte Absprachen innerhalb der Traumanetzwerke® DGU bei einem MANV konnten im Gegensatz zum Aspekt der Absprache mit Behörden hier von zwei Dritteln der Befragten mit „ja“ beantwortet werden (*n* = 56, 66 %), 17 % beantworteten die Frage mit „nein“, 17 % waren Absprachen innerhalb des eigenen TraumaNetzwerks® DGU unbekannt (Abb. 8).

Eine weitere Frage adressierte die Einschätzung zum Wege- und Raumkonzept innerhalb der eigenen Klinik. Die Hälfte der Befragten (*n* = 39, 47 %) gab an, dass das eigene Wege- und Raumkonzept bei einem MANV mit Terrorhintergrund nicht durch Absperrmaßnahmen geschützt und umgesetzt werden kann. Dies gelänge bei nur einem Drittel (*n* = 28, 34 %), 19 % sei die Möglichkeit einer Umsetzung unbekannt.

Folgende Ergebnisse konnten aus dem Themenkomplex gewonnen werden, der sich thematisch mit stattgehabten Übungen eines MANV in den Kliniken, deren Frequenz und Art sowie Ausgestaltung der Übungen befasste:

Die Frage nach der Häufigkeit der durchgeführten Übungen beantworteten 41 % (*n* = 35/84) der Befragten mit „mehrfach“, 27 % (*n* = 23) mit „einmalig“ und 31 % (*n* = 26) mit „nie“. Ein Fragebogen wurde ohne eine Angabe ausgefüllt. Zusätzlich wurde in dieser Frage weiterführend als Antwortmöglichkeit offeriert, ob diese Übungen mit oder ohne terroristischen Hintergrund inszeniert wurden. Diesbezüglich haben 74 % (*n* = 29/39) in ihren Klinikeinrichtungen Szenarien ohne Terrorhintergrund geübt, im Gegensatz zu 26 % (*n* = 10) mit Terrorhintergrund. Allerdings zählten wir 21 Fragebogen ohne Angabe.

**Figure 9:**
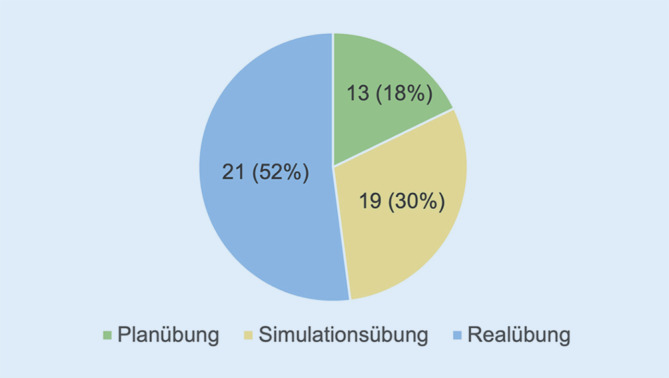

Weiterhin wurde die Art der Durchführung der jeweiligen Übungen abgefragt:

Eine „Realübung“ haben 52 % (*n* = 38/73) der Befragten durchgeführt, 30 % (*n* = 22) als „Simulationsübung“ und 18 % (*n* = 13) als „Planübung“ (Abb. 9). 5 Fragebogen (6 %) wurden ohne eine Antwort nicht berücksichtigt.

- Als „Realübung“ definieren wir Vollübungen der jeweiligen Kliniken mit Einbindung aller notwendigen Ressourcen, wie z. B. Simulationspatienten*innen, um reale Versorgungsprozesse abbilden zu können.
- „Simulationsübungen“ im Gegensatz umfassen vorgefertigte dynamische Szenarien, in welchen die Ressourcen „Personal, Material und Zeit“ möglichst realitätsnah dargestellt werden sollen.
- „Planübungen“ wiederum zielen v. a. darauf ab, die Handlungskompetenzen von Führungsstrukturen zu beüben und zu erweitern, indem vorgefertigte Szenarien modellhaft und eher statisch durchgespielt werden.

Bei 58 % (*n* = 34/59) der Befragten, die eine Übung zu einem MANV/*Terror*MANV in der Vergangenheit durchführen konnten, erfolgte diese nach entsprechender Ankündigung. Nur 16 % (*n* = 13) übten ihre Szenarien ohne eine vorherige Ankündigung.

**Figure 10:**
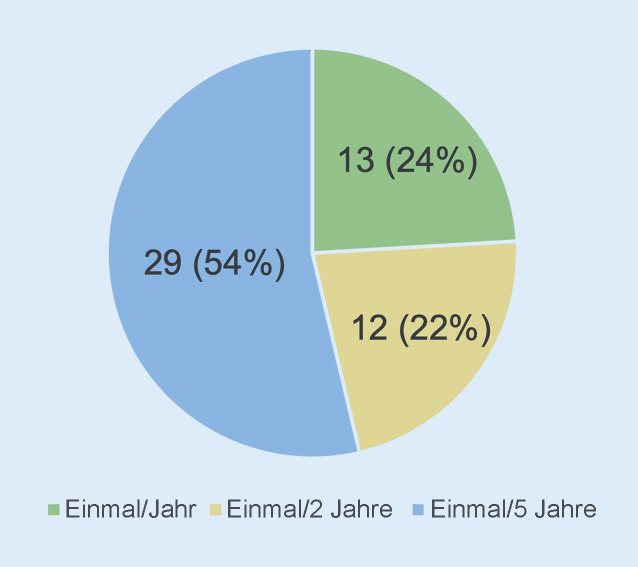

Hinsichtlich des zeitlichen Rhythmus der Übungen bzw. der zeitlichen Abstände zwischen den Übungen in den Kliniken gab die Mehrheit mit 54 % (*n* = 29/54) Übungen mit einem Intervall von einer Übung alle 5 Jahre, 22 % (*n* = 12) alle 2 Jahre und 24 % (*n* = 13) einmal pro Jahr an (Abb. 10).

**Figure 11:**
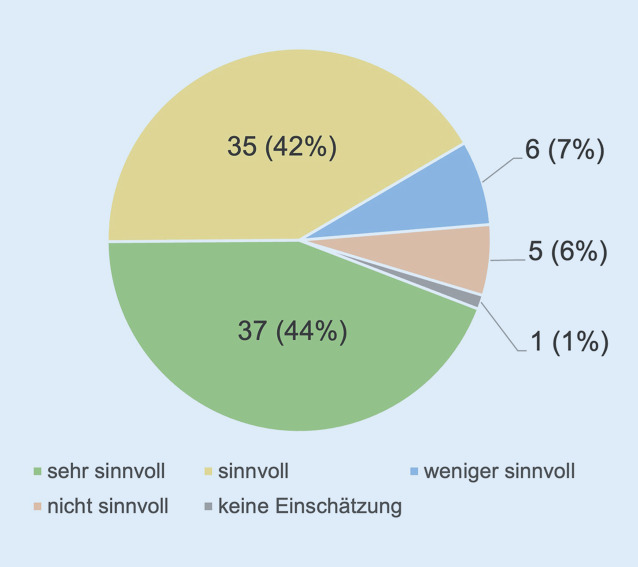

„Terror preparedness“-Kriterien als Zertifizierungskriterien von Traumazentren: Zwei Fragen adressierten die Einschätzung der Befragten, ob die Überprüfung von Kriterien zur „Terror preparedness“ zur Rezertifizierung der TraumaNetzwerke® DGU Eingang finden sollte (Abb. 11). Darauf aufbauend wollten wir von den Befragten wissen, welche Kriterien in diesem Zusammenhang überprüft werden sollten.

Es stellte sich heraus, dass 44 % (*n* = 37) der Befragten eine Überprüfung der Terror preparedness zur Rezertifizierung als „sehr sinnvoll“ erachten, 42 % (*n* = 35) bewerteten dies als „sinnvoll“. Nur 7 % (*n* = 6) bzw. 6 % (*n* = 5) hielten eine Überprüfung für „weniger sinnvoll“ oder „nicht sinnvoll“.

Hinsichtlich der zu prüfenden Kriterien wurden den Befragten im Fragebogen die Antwortmöglichkeiten „KAEP“, „Lagebeurteilung“, „Personalkonzept“, „Materialkonzept“ und „geschultes Personal“ zur Auswahl gestellt.

Das Kriterium „KAEP“ wurde zu 100 % (*n* = 85) befürwortet, gefolgt vom „Personalkonzept“ mit 95 % (*n* = 81), „Materialkonzept“ mit 94 % (*n* = 80) sowie „Lagebeurteilung“ und „geschultes Personal“ mit je 82 % (*n* = 69).

## Diskussion

In unserer Publikation greifen wir das Thema „Sicherheit in und an der Klinik“ auf. Die Zielsetzung bestand darin, durch eine Literatursichtung und eine Befragung der Teilnehmer*innen der 3. Notfallkonferenz der DGU zu beleuchten, inwieweit Kliniken auf mögliche Sicherheitsrisiken vorbereitet sind und welche Bemühungen zur Daseinsberechtigung bisher dahingehend unternommen wurden.

Hierbei geht es konkret um den Schutz einer kritischen Infrastruktur in unserer Gesellschaft, die potenziell durch terroristische Aktivitäten bedroht wird. Da es in solchen Szenarien zu einem Massenanfall von Verletzten mit sehr dynamischen Lagen kommen kann, ist ein ausgeklügeltes und belastungsstabiles Sicherheitskonzept mit einem Zusammenspiel von verschiedenen Akteuren gefragt, um die Funktionalität der Kliniken aufrechtzuerhalten.

### Bestandsaufnahme

Die Verpflichtung zur Gefahrenabwehr für die kritische Infrastruktur „Klinik“ liegt hoheitlich bei den Bundesländern und ist dementsprechend rechtlich auch in den Katastrophenschutzgesetzen oder Krankenhausgesetzten der Länder festgelegt. Ein wichtiges Instrument ist das Vorhalten einer KAEP [25].

In unserer Befragung zeigte sich, dass eine KAEP bei 84 % der Befragten vorlag. Hiermit scheint sich dieses Instrument zur Etablierung von Maßnahmen zur Sicherheit an und in deutschen Kliniken folgerichtig durch verschiedene Publikationen und Lenkungsgruppen zur Erstellung von Handlungsempfehlungen durchgesetzt zu haben.

Bei knapp zwei Dritteln der Befragten wurde die KAEP innerhalb der letzten 3 Jahre zuletzt aktualisiert, sodass man an dieser Stelle interpretieren darf, dass u. a. terroristische Vorkommnisse der jüngsten Vergangenheit eine entsprechende Reaktion und nötige Anpassungen bewirkt haben.

89 % der Befragten gaben ein bestehendes Alarmierungskonzept in ihrer Klinikstruktur an, das bei 60 % der Befragten überwiegend „automatisiert und abgestuft“ funktionierte. Im Gegensatz zu einer „individuellen und manuellen“ Alarmierung liegt der Vorteil bei einem computergestützten, automatisierten System in der gleichzeitigen Alarmierung einer großen Personenzahl und der parallelen Nutzung unterschiedlicher Alarmierungswege (z. B. Festnetz, Mobilfunk, E‑Mail, SMS), während ein individuell-manuelles Konzept Personal bindet und darüber hinaus auch zeitaufwendig ist. Deshalb scheint sich hier die technisch unterstützte Alarmierung durchgesetzt haben. Nichtsdestotrotz sollte bei technischen Störungen oder sogar Ausfällen auf alternative IT- und Kommunikationsmittel im Notfall zurückgegriffen werden können [24].

Neben der Aktualisierung der KAEP spielt auch die Kommunikation zu den Mitarbeitern bezüglich Aktualisierungen und Neuerungen von Inhalten eine wichtige Rolle. Unsere Befragung zeigte hier auf, dass etwa die Hälfte der Befragten (49 %) keine Veranstaltungen zu diesem Thema durchführte. Gerade als Möglichkeit zur Sensibilisierung des hausinternen Personals als auch zur aktiven Gestaltung sowie kritischen Diskussion der Prozesse erachten wir solche Veranstaltungen in regelmäßigen Intervallen als sinnvoll. Als Leitfaden zur Frequenz könnte man vergleichend die von uns erhobene Statistik nennen, dass Besprechungen zur „normalen Notfallversorgung“ in deutschen Traumazentren überwiegend mindestens einmal im Quartal stattfinden, wobei damit einmal jährlich derartige Besprechungen für den Schwerpunkt KAEP genutzt werden könnten.

Eine zentrale Fragestellung unserer Publikation betraf das Vorhandensein eines innerklinischen Sicherheitskonzeptes. Diese Bestandsaufnahme zeigte, dass doch immerhin ein signifikanter Anteil der Befragten kein Sicherheitskonzept in der Klinik vorweisen konnte (38 %) oder zumindest Unklarheit über solch ein Konzept bestand (28 %). Korrespondierend konnten wir feststellen, dass die Hälfte der Befragten nicht mit einem Sicherheits- oder Wachdienst kooperierte.

Dies sind Indizien, dass das Thema „Sicherheit in der Klinik“ konzeptionell noch nicht den breitflächigen Durchbruch erreicht und im alltäglichen Betrieb keine routinemäßige Umsetzung genießt. Andererseits könnte diese Tatsache dem Umstand geschuldet sein, dass die interne Risikoanalyse gemäß der KAEP nicht zu einer Bewertung geführt hat, die eine Zusammenarbeit mit einem Sicherheitsdienst erfordert.

Aus den Erfahrungen, die wir durch Begleitung von Großschadensübungen (z. B. „BWTEX“ 2019 in Baden-Württemberg, größte interdisziplinäre (Antiterror‑)Übung der Sicherheitsbehörden in der Geschichte der Bundesrepublik Deutschland) gewinnen konnten, resultiert jedoch die Erkenntnis, dass in solch dynamischen und komplexen Szenarien wie z. B. einem MANV oder *Terror*MANV klare Absprachen mit den lokalen Behörden getroffen werden müssen, damit beim Eintreten von besonderen Ereignissen Personal zur Sicherung der Klinik und zum „Clearing“ von eintreffenden Patienten benannt ist. Dies ist ein Faktor, der durch das klinikeigene Personal nicht gewährleistet werden kann.

Ebenso wichtig sind die Absprachen innerhalb der Traumanetzwerke, da beim Auftreten von bestimmten Ereignissen, wie sie schon in der Vergangenheit aufgetreten sind (z. B. Großschadenereignisse wie Rammstein 1988 und Eschede 1998), übergeordnete Strukturen aktiviert werden müssen, um einen strukturierten Abstrom von einer großen Anzahl an Patienten aus den Lagen zu gewährleisten [16, 19]. Im Vergleich zu einem *Terror*MANV, der eine größere Dynamik aufweist, sind diesbezüglich Absprachen, inklusive Organisation von Sekundärverlegungen, umso wichtiger [1]. Hier zeigt sich in der Befragung der Trend, dass die eine Hälfte der Befragten einen zentralen Ansprechpartner im jeweiligen Traumanetzwerk hat, während die andere Hälfte jedoch keine zentrale Kontaktmöglichkeit besitzt oder diese unbekannt ist. In diesem Punkt zeigt sich ein klarer Informationsbedarf innerhalb der Traumanetzwerke, der durch geeignete Maßnahmen wie z. B. netzwerkinterne Informationsveranstaltungen generiert werden kann.

Weitere wichtige Aspekte hinsichtlich der vorbereitenden Maßnahmen zum Thema „Sicherheit an und in der Klinik“ sind der Umgang mit sowie die Vorbereitung auf mögliche CBRN-Bedrohungen. Obwohl in der jüngeren Vergangenheit keine terroristischen Angriffe in Europa mit biologischen, nuklearen, chemischen oder radioaktiven Kampfmitteln oder -stoffen stattgefunden haben, sollten diese weiterhin in die Risikoanalysen der KAEP einbezogen werden.

Dementsprechend verfügt die Mehrheit der Befragten in den Kliniken über keinerlei Dekontaminationseinheit (71 %). Die Verfügbarkeit oder der Anschluss an eine Dekontaminationseinheit als eine Maßnahme zur strukturierten Bewältigung solcher Bedrohungen sollte dennoch kritisch geprüft und reevaluiert werden, um den besonderen Herausforderungen bei CBRN-Notfällen begegnen zu können [20]. Eine Option zum Erwerb einer entsprechenden Expertise kann hier möglicherweise der intensivere „Schulterschluss“ mit der Bundeswehr sein, die mit ihrer Expertise im Umgang mit „ABC-Bedrohungen“ sowie mit der technischen Ausstattung als wertvoller Partner in einer Beraterfunktion zur Seite stehen kann.

### Bisherige Bemühungen und Ausblick

In der Betrachtung der Bemühungen oder bisherigen Vorbereitungen der Kliniken zum Thema Sicherheit muss man feststellen, dass nur 34 % der Klinikeinrichtungen ein Sicherheitskonzept besitzen. Dies scheint als „Basisindikator“ für die Thematik ein recht geringer Wert zu sein. Hier müssen die Ursachen weiter untersucht werden. Mögliche Gründe für dieses Ergebnis könnten finanzielle Belange sein oder eine als nachrangig eingeschätzte Notwendigkeit zur Implementierung solcher Konzepte.

Was in der Auswertung der Fragen zum Themenkomplex „Vorhandensein Sicherheitskonzept“ evident war, dass beinahe einem Drittel der Befragten „unbekannt“ war, ob ein Sicherheitskonzept oder auch Absprachen mit Sicherheitsbehörden wie z. B. der Polizei, Feuerwehr, Sicherheitsdiensten bei einem MANV vorliegen. Dies verdeutlicht, dass der Stellenwert der Kommunikation und der Grad der Vernetzung mit öffentlichen Behörden deutlich gesteigert werden müssen, um hier in einem etwaigen Szenario klare Strukturen und Zuständigkeiten zu schaffen [13, 26]. Absprachen, die nicht bekannt und kommuniziert sind, werden zum einen nicht umgesetzt und zum anderen bei geringer Compliance unnötigerweise hinterfragt und Umsetzungen durch Rückfragen verzögert.

Wiederum deutlich klarer scheinen die Absprachen innerhalb der TraumaNetzwerke DGU® zu sein, da hier eine klare Mehrheit von 66 % der Befragten konkrete Absprachen bestätigen konnte. Dies zeigt, dass der fachbezogene Austausch in der Regel und auf der Basis der gut etablierten Netzwerkstrukturen in der Regel unkompliziert ist und leichter fällt, sowie diese deutlich häufiger getroffen werden als mit „entfernteren“ Institutionen.

Der konkrete Bedarf an einer Steigerung der Vernetzung zu öffentlichen Behörden ist jedoch klar abzuleiten, denn immerhin beschreiben 47 % der Befragten, dass ihr vorliegendes Raumkonzept bei einem MANV/*Terror*MANV nicht umgesetzt bzw. geschützt werden kann. Dies impliziert, dass weitere Schulungen/öffentliche Veranstaltungen erfolgen sollten, um Klinikleitungen und den Hauptverantwortlichen Möglichkeiten aufzuzeigen, ihr Anfahrts- und Sicherheitskonzept außerhalb der Klinik sowie das innerklinische Wege- und Raumkonzept zu optimieren.

Im Bereich des Anfahrtkonzeptes könnte man z. B. getrennte Zu- und Abfahrtswege der Einsatzfahrzeuge im Sinne eines Kreisverkehrs definieren oder die Regelung der Verkehrsführung mit Ausschilderung durch Polizei oder lokale Feuerwehr aufführen.

Ein wesentliches Element zur Vorbereitung auf einen MANV oder *Terror*MANV ist das Training und Einstudieren von Abläufen, um Schwächen oder Ungereimtheiten in eigenen Abläufen erkennen zu können. Die besonderen Zeitachsen der Patientenströme und zu erwartenden Verletzungsmuster wurden bereits in verschiedenen Publikationen ausgeführt [1, 3, 4, 8, 9].

Dazu kommt, dass gerade das Zusammenspiel mit öffentlichen Behörden aufgrund der verschiedenen Zuständigkeiten, Kommunikationsarten und -mittel und der strategischen Herangehensweisen einer ausgeklügelten, aber klaren Interaktion bedarf.

Nachdem in der Vorbereitung für die Fußballweltmeisterschaft 2006 kurzzeitig viele Anstrengungen von Krankenhäusern und lokalen Behörden unternommen wurden, um die Versorgung von Großschadensereignissen zu verbessern [15, 23], zeigen sich in unserer aktuellen Auswertung, dass es bei der Umsetzung und Durchführung von Übungsvorhaben in den letzten Jahren verschiedene Herangehensweisen gibt.

Belegt wird diese Feststellung durch die vorliegende Auswertung, dass nur knapp die Hälfte aller Befragten in ihren Wirkstätte eine Übung innerhalb der letzten 5 Jahre miterlebt hat. Ein Viertel der Befragten berichtet im Gegensatz dazu einmal pro Jahr eine Übung in der eigenen Klinik. Diese Heterogenität könnte dadurch erklärt werden, dass es Kliniken gibt, die entweder aufgrund einer intrinsischen Motivation deutlich häufiger Zeit und Ressourcen für solche Vorhaben aufwenden oder durch politische Unterstützung ggf. eine stärkere Unterstützung erfahren, mit Bereitstellung von finanziellen Mitteln oder strikteren Vorgaben innerhalb der jeweiligen Landeskatastrophenschutzgesetze. Dass die Hälfte aller Übungen als „Realübung“ durchgeführt wurde, ist wiederum als sehr positiv zu bewerten, da diese Art von Übungsmuster die betroffenen Kliniken personell und materiell am stärksten belastet und damit auch in der Lage ist, etwaige bestehende Defizite realitätsnah und deutlich aufzuzeigen. So konnte z. B. im Rahmen der Realübung „BWTEX“ im Oktober 2019 bereits eine konstruktive Auswertung der Übung mit anschließender Aufarbeitung durchgeführt werden [21].

Es zeigt sich, dass v. a. Übungsvorhaben ohne Terrorhintergrund (76 %) durchgeführt worden sind. Hier sollte zukünftig das Übungsspektrum erweitert werden, da der Anstieg von terroristischen Aktivitäten in Europa mit Schuss- und Explosionsverletzungen ein taktisches Umdenken auf allen Ebenen in der Bewältigung solcher Szenarien im Gegensatz zu einem MANV erfordert [2, 8].

## Zusammenfassung

Zusammenfassend stellen wir fest, dass in deutschen Traumazentren Maßnahmen zur Gewährleistung von Sicherheit an und in der Klinik durchgeführt werden. Auf Planungsebene sehen wir die Kliniken hinsichtlich der KAEP anhand der gewonnenen Daten grundsätzlich gut aufgestellt.

## Fazit für die Praxis

- Aus unserer Sicht ergehen dabei folgende Empfehlungen bezüglich der weiteren Umsetzung:
  Eine Aktualisierung der KAEP sollte aus unserer Sicht jährlich erfolgen, um die eigene Risikoanalyse der zunehmend kürzeren Halbwertszeit von politischen Ereignissen und Veränderungen anzupassen,
  Intensivierung der Kooperationen mit Sicherheits- und Wachdiensten und damit Erstellung eines Sicherheitskonzeptes,
  Anpassung der Alarmierungskonzepte an die technischen Möglichkeiten der heutigen Zeit unter Vorhaltung von störunanfälligen Rückfallebenen,
  Berücksichtigung von CBRN-Bedrohungen in der Risikoanalyse und Etablierung von Gegenmaßnahmen vor Ort (z. B. Schutzausrüstung, Dekontaminationseinheiten).
  Standardisierte Vorgaben und Zielsetzungen hinsichtlich der Übungsvorhaben an und in deutschen Kliniken.
- Die genannten Empfehlungen sollten im Rahmen der Daseinsvorsorge der deutschen Kliniken als Kriterien im Sinne einer „Terror preparedness“ überprüft werden.
- Die klare Mehrheit der Befragten in unserer Umfrage erachtet eine derartige Überprüfung im Rahmen der Rezertifizierung TZ/TNW als sinnvolle Maßnahme.
- Im Einklang mit den Qualitätsrichtlinien und Zertifizierungskriterien TNW im *Weißbuch Schwerverletztenversorgung* der DGU (3., erweitere Auflage, 2019) sollte der erforderliche Nachweis innerhalb von 3 Jahren nach der letzten Überprüfung erbracht werden.
- Dazu erscheint das Thema Sicherheit an und in Kliniken im Hinblick auf die weiterhin latente Terrorbedrohung in Deutschland noch nicht umfänglich bearbeitet und wahrgenommen, und es besteht noch Optimierungspotenzial.
